# Taiwanin E Induces Cell Cycle Arrest and Apoptosis in Arecoline/4-NQO-Induced Oral Cancer Cells Through Modulation of the ERK Signaling Pathway

**DOI:** 10.3389/fonc.2019.01309

**Published:** 2019-12-17

**Authors:** Shih-Hao Wang, Hsi-Chin Wu, Khan Farheen Badrealam, Yueh-Hsiung Kuo, Yun-Peng Chao, Hsi-Hsien Hsu, Da-Tian Bau, Vijaya Padma Viswanadha, Yi-Hui Chen, Pei-Jei Lio, Chung-Jen Chiang, Chih-Yang Huang

**Affiliations:** ^1^Department of Otolaryngology, Ditmanson Medical Foundation, Chiayi Christian Hospital, Chiayi, Taiwan; ^2^Department of Biotechnology, Asia University, Taichung, Taiwan; ^3^School of Medicine, China Medical University, Taichung, Taiwan; ^4^Graduate Institute of Biomedicine, China Medical University and Hospital, Taichung, Taiwan; ^5^Department of Chinese Pharmaceutical Sciences and Chinese Medicine Resources, China Medical University, Taichung, Taiwan; ^6^Chinese Medicine Research Center, China Medical University, Taichung, Taiwan; ^7^Department of Chemical Engineering, Feng Chia University, Taichung, Taiwan; ^8^Division of Colorectal Surgery, Mackay Memorial Hospital, Taipei, Taiwan; ^9^Mackay Medicine, Nursing and Management College, Taipei, Taiwan; ^10^Department of Biotechnology, Bharathiar University, Coimbatore, India; ^11^Department of M-Commerce and Multimedia Applications, Asia University, Taichung, Taiwan; ^12^Department of Medical Laboratory Science and Biotechnology, China Medical University, Taichung, Taiwan; ^13^Cardiovascular and Mitochondria Related Diseases Research Center, Hualien Tzu Chi Hospital, Hualien, Taiwan; ^14^Center of General Education, Buddhist Tzu Chi Medical Foundation, Tzu Chi University of Science and Technology, Hualien, Taiwan; ^15^Department of Medical Research, China Medical University Hospital, China Medical University, Taichung, Taiwan

**Keywords:** Taiwanin E, oral cancer, apoptosis, cell cycle arrest, therapeutics

## Abstract

Taiwanin E is a bioactive compound extracted from *Taiwania cryptomerioides* Hayata. In this research endeavor, we studied the anti-cancer effect of Taiwanin E against arecoline and 4-nitroquinoline-1-oxide-induced oral squamous cancer cells (OSCC), and elucidated the underlying intricacies. OSCC were treated with Taiwanin E and analyzed through MTT assay, Flow cytometry, TUNEL assay, and Western blotting for their efficacy against OSCC. Interestingly, it was found that Taiwanin E significantly attenuated the cell viability of oral cancer cells (T28); however, no significant cytotoxic effects were found for normal oral cells (N28). Further, Flow cytometry analysis showed that Taiwanin E induced G1cell cycle arrest in T28 oral cancer cells and Western blot analysis suggested that Taiwanin E considerably downregulated cell cycle regulatory proteins and activated p53, p21, and p27 proteins. Further, TUNEL and Western blot studies instigated that it induced cellular apoptosis and attenuated the p-PI3K/p-Akt survival mechanism in T28 oral cancer cells seemingly through modulation of the ERK signaling cascade. Collectively, the present study highlights the prospective therapeutic efficacy of Taiwanin E against arecoline and 4-nitroquinoline-1-oxide-induced oral cancer.

## Introduction

Over the years, complementary and alternative medicine therapies have garnered great attention all across the globe (1, 2). Accumulating evidences has highlighted the efficacy of various small molecule compounds/phytochemicals as effective therapeutic entities against various diseases and pathological conditions, including cancer (3–6). Previous reports from our group have ascertained the potential of several bioactive compounds against cancer together with providing an insight into their mechanisms of action (7–11).

Taiwania (*Taiwania cryptomerioides* Hayata) represents one of the most economically relevant plant species endemic to Taiwan. Numerous bioactive compounds have been derived from this plant species. Many of them have been demonstrated to exhibit potent activity against bacteria, fungi, termites, mites, and cancers (12–15). To this end, recently, we have provided convincing evidence for the efficacy of Taiwanin A against arecoline and 4-nitroquinoline-1-oxide-induced oral cancer (16–18). Nevertheless, to the best of our knowledge, the effect of Taiwanin E against oral cancer and the underlying mechanism remains poorly understood.

Despite advancement in the allied field of biomedical sciences, the repercussions that may arise from cancer represent a significant human toll. According to statistics, globally, oral cancer is amongst 10 most common cancers. Oral squamous cell carcinoma (OSCC) is the most common malignant epithelial neoplasm that can afflict the oral cavity. It is thought that more than 90% malignancies arising from the head and neck tissue section are OSCC (19). Despite the availability of treatment strategies, including surgery, radiation, and chemotherapy, the overall survival rate of patients remains poor (20, 21). Taking these into consideration, in the current research endeavor, we have studied the effect of Taiwanin E against oral cancer and elucidated the underlying mechanism for their efficacy against oral cancer.

Interestingly, it was found that Taiwanin E significantly attenuated the cell viability of oral cancer cells (T28) in a dose- and time-dependent fashion; nevertheless, no cytotoxic effects were found for normal oral cells (N28). Moreover, it was observed that Taiwanin E induces G1 cell cycle arrest in T28 cells, as was evident through Flow cytometry studies, and, further, Western blot analysis suggested that Taiwanin E considerably downregulated cell cycle regulatory proteins and activated p53, p21, and p27 proteins. In addition, TUNEL staining showed that Taiwanin E induced apoptosis in T28 oral cancer cells. Furthermore, it was found that the cell survival proteins, such as p-PI3K, p-Akt, and the antiapoptotic protein Bcl-xL, were considerably reduced following treatment with Taiwanin E; nevertheless, the pro-apoptotic proteins, such as Bax, Cyt C, and c Cas 3, were, however, considerably enhanced. Further, understanding the underlying intricacies; mechanistically, it was found that Taiwanin E modulated the expression of ERK and resulted in cellular apoptosis in T28 oral cancer cells. Taken together, the data convincingly ascertained the promising candidature of Taiwanin E against oral cancer.

## Materials and Methods

### Chemicals and Reagents

All chemicals and reagents were procured from Sigma Aldrich Co. (MO, USA) unless otherwise mentioned.

### Purification of Taiwanin E

Taiwanin E was obtained from freshly cut wood of *Taiwania cryptomerioides* Hayata. The procedures for isolation, purification, and characterization of Taiwanin E was performed following our previously published reports with slight modifications (22, 23). Finally, the as-purified Taiwanin E was dissolved in DMSO, filtered through 0.22 μm fluoropore filter (Millipore, MA, USA), and employed for subsequent studies.

### Establishment of Cell Model for Oral Cancer

An OSCC model was established following the protocol described in our previous studies (16, 17). Basically, carcinogenesis was induced in C57BL/6J Narl male mice by daily oral administration of 0.5 mg/mL arecoline (Sigma Aldrich, MO, USA) and 0.2 mg/mL of 4-NQO (Sigma Aldrich, MO, USA) for 28 days. Thereafter, primary oral squamous carcinoma cells were derived from tumor (T28) tissue following 28 weeks of administration. In addition, primary oral squamous cells were also derived from a paired control group, i.e., non-tumor normal (N28), tissue, and these were used as normal control cells.

All the animal experimentation protocols performed in the study were strictly in accordance with the Animal Care and Use Committee of the China Medical University, Taichung, Republic of China (Taiwan).

N28 and T28 cells were cultured in Dulbecco's minimum essential medium (D7777) (Sigma Aldrich, MO, USA) supplemented with 10% charcoal-treated FBS (Characterized Fetal Bovine Serum, HyClone Inc., Utah, USA), 1% penicillin/ streptomycin (Invitrogen Corp., California, USA), L-Glutamine, and NaHCO_3_.

For treatment, cells were seeded in triplicate in cell culture plates and treated with varying concentrations of Taiwanin E (0, 1, 5, and 10 μM) for different time intervals (0, 3, 6, 12, 24, and 48 h), and thereafter, the cells were harvested and analyzed for respective parameters. For activator experiments, cells were treated with Taiwanin E (10 μM) and co-treated with increasing concentrations of ERK activator (0.5, 1.0, 2.5, and 5.0 μM) for 24 h. Thereafter, they were analyzed by MTT assay and Western blotting, following the standard procedures as described below.

### Cell Viability Assay

Cell viability was assessed as reported in our previous studies (16). Basically, cells were seeded in triplicate in cell culture plates and treated with increasing concentrations of Taiwanin E (0, 1, 5, and 10 μM) for a 24 h time interval. In addition, cells were treated with Taiwanin E (10 μM) for different time intervals (0, 3, 6, 12, 24, and 48 h). Following incubation with Taiwanin E for varying concentrations and time periods, the cells were thereafter treated with 0.5 mg/mL of MTT reagent and incubated for 4 h in dark conditions. Finally, the formazan crystals were dissolved in 500 μL of DMSO (dimethyl sulfoxide), and the absorbance was acquired at 570 nm on a multi-well ELIZA plate reader. Cell viability was represented as the percentage of control.

### Cell Cycle Analysis

Cell cycle study was performed with flow cytometry. Briefly, T28 oral cancer cells (1 × 10^5^ cells/well) were seeded in tissue culture plates; thereafter, they were treated with varying doses of Taiwanin E (0, 1, 5, and 10 μM) for 24 h and treated with Taiwanin E (10 μM) for different time intervals (0, 3, 6, 12, 24, and 48 h). Following treatment, cells were harvested and subsequently fixed in chilled ethanol overnight at 4°C. Consequently, cells were washed with PBS, centrifuged at 600 ×g for 5 min and thereafter resuspended in PBS containing 10 mg/ml RNase A and incubated with 30 mg/ml of PI for 30 min at RT. Finally, the samples were acquired with flow cytometry (Becton Dickinson, CA, USA).

### Western Blot Analysis

Western blot was executed as reported previously (16, 24). In brief, cells were lysed in RIPA lysis buffer containing 50 mM Tris, pH 7.5, 150 mM NaCl, 1% NP-40, 0.1% SDS, 0.5% sodium deoxycholate, phosphatase inhibitor cocktail, and a proteinase inhibitor cocktail and thereafter centrifuged at 12,000 × g for 30 min at 4°C to obtain the cell lysate (25). After this, the supernatants were obtained, and protein concentration was estimated through a Bio-RAD protein quantification reagent following the Bradford method. Thereafter, the samples were resolved through 8–12% sodium dodecyl sulfate polyacrylamide gel electrophoresis (SDS-PAGE) and blotted onto polyvinylidene difluoride (PVDF) membranes (Millipore, MA, USA). The membranes were thereafter blocked with blocking solution (5% skimmed milk in TBST) for 1 h at RT. Following the blocking steps, the membranes were washed with TBST thrice and finally incubated with primary antibodies against β-actin, Cyclin B1, Cyclin D1, Cyclin E, p21, p27, JNK, p-ERK, p-JNK, p38, Cytochrome C, Bcl-xL, and Bax from Santa Cruz (CA, USA); pS473-Akt, p-PI3K, cleaved caspase-3, pP53, and p-p38 from Cell Signaling (CA, USA); and ERK from BD Biosciences (CA, USA). Following the washing procedures, the membranes were then subsequently incubated with secondary antibodies for 1 h at RT. Finally, the Antigen–Antibody molecular intricacies were assessed with enhanced chemiluminescence (ECL) horseradish peroxidase (HRP) substrate (Millipore, MA, USA), and the signals were acquired by LAS 3,000 imaging system (Fujifilm, Tokyo, Japan). Membranes were stripped and reprobed with other antibodies for the subsequent detection of other proteins.

### TUNEL Assay

Cellular apoptosis was analyzed by *in situ* terminal deoxynucleotide transferase-mediated dUTP nick end-labeling (TUNEL) assay following manufacturer-recommended procedures (26). In brief, cells were cultured in 24-well culture plates and treated with Taiwanin E, and, thereafter, the cells were harvested, washed with 1×PBS (Gibco BRL, Paisley, UK), and incubated with TUNEL assay reagents at RT for stipulated time intervals. After this, the samples were examined with flow cytometry (Becton Dickinson, CA, USA).

### Statistical Analysis

Results are represented as mean ± SD. Statistical analysis was done with Graph Pad Prism5 statistical software (Graph-Pad, CA, USA). Multiple comparisons were assessed through ANOVA. *p* Values of ≤ 0.05 were regarded as statistically significant. All experiments were performed in triplicate in a blinded manner. Results were quantified with Image J software (NIH, Bethesda, MD, USA) and processed through Adobe Photoshop.

## Results

### The Effect of Taiwanin E on Cell Viability and the Cell Cycle Process of Oral Cancer Cells Following Dose-Dependent Treatment

**Figure 1:**
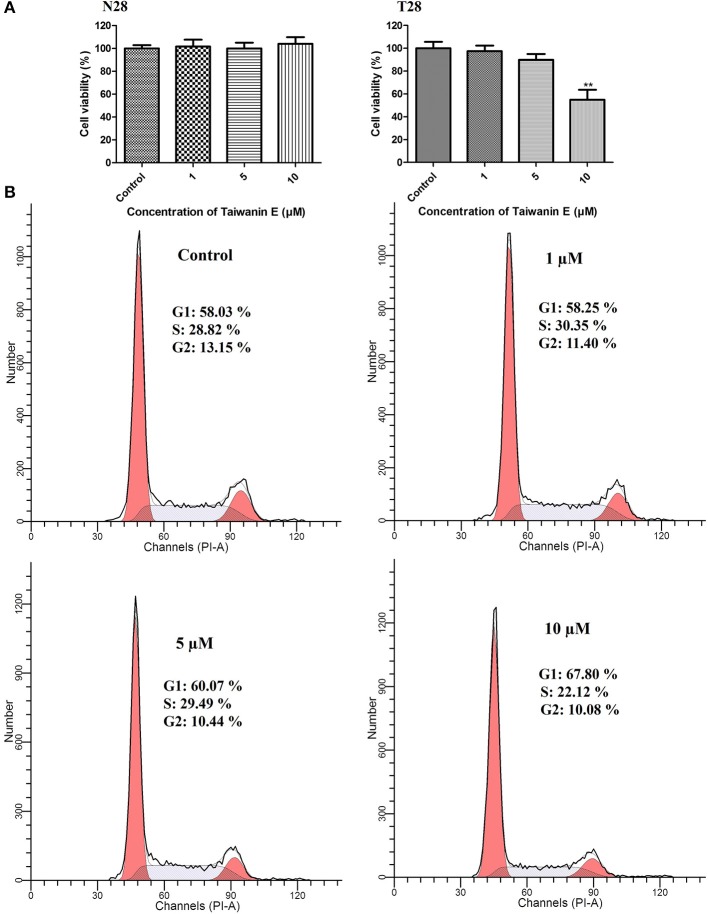
Effect of Taiwanin E on cell viability and cell cycle progression of oral cells. Normal oral cells N28 and Cancer oral cells T28 were treated with varying concentrations of Taiwanin E (0, 1, 5, and 10 μM) for 24 h. The cell viability of N28 cells and T28 cells was assessed with MTT assay **(A)**. **p < 0.01 represents significant differences compared with control. T28 cells were treated with varying concentrations of Taiwanin E (0, 1, 5, and 10) μM for 24 h, and the DNA content was assessed through flow cytometry. Results were represented as percentages of the cell population in G1, S, and G2 phases of the cell cycle **(B)**.

Normal oral cells N28 and cancer cells T28 were treated with increasing concentrations of Taiwanin E for a 24 h time interval; thereafter, cell viability was ascertained by MTT assay. MTT data showed that Taiwanin E exhibited cytotoxic effects on T28 oral cancer cells, especially at 10 μM concentration, whereas no cytotoxic effects were observed for N28 normal oral cells (Figure 1A). In addition, it was observed that Taiwanin E exhibited significant cytotoxicity against other squamous cell carcinoma cell lines SCC9 and SCC25 (data not shown). Considering the results, we further examined whether Taiwanin E treatment could modulate cell cycle progression in oral cancer cells T28. To this end, cells were treated with increasing concentrations of Taiwanin E for 24 h; thereafter, they were analyzed through flow cytometry. As evident from Figure 1, it was found that there were considerable increase in the G1 population (60.07% at 5 μM, 67.80% at 10 μM) in Taiwanin E-treated T28 cells as compared to control cells, wherein only 58.03% of cells were in the G1 population (Figure 1B). The data from the study suggested an accumulation in the G1 phase of the cell cycle following treatment with Taiwanin E.

### The Effect of Taiwanin E on Cell Viability and the Cell Cycle Process of Oral Cancer Cells Following Time-Dependent Treatment

**Figure 2:**
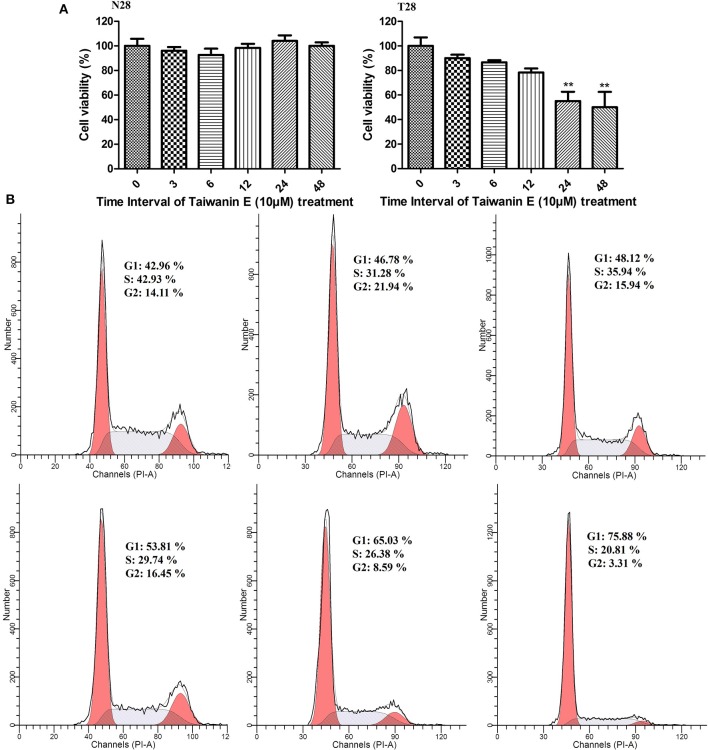
Effect of Taiwanin E on cell viability and cell cycle progression of oral cells. Normal oral cells N28 and cancer oral cells T28 were treated with Taiwanin E (10 μM) for different time intervals. The cell viability of N28 cells and T28 cells was assessed with MTT assay **(A)**. **p < 0.01 represents significant differences compared with control. T28 cells were treated with Taiwanin E (10 μM) for different time interval, and the DNA content was assessed through flow cytometry. Results were represented as a percentage of the cell population in G1, S, and G2 phases of the cell cycle **(B)**.

Further, N28 and T28 cells were treated with Taiwanin E (10 μM) for different time periods from 0 to 48 h, and cell viability was determined through MTT assay; interestingly, it was observed that Taiwanin E exhibited cytotoxic effects on T28 cells in a time-dependent manner. However, no such effects were observed for normal N28 oral cells (Figure 2A). Further, we also determined the effect of treatment of Taiwanin E for different time interval (0–48 h) on the cell progression; it was found that there were considerable increases in the G1 population in a time-dependent fashion (Figure 2B) (27).

Collectively, these results suggested that Taiwanin E exhibited cytotoxic manifestations toward tumor oral cells T28 in a dose- and time-dependent fashion; however, no cytotoxic effects were observed for normal oral cells N28. Moreover, Taiwanin E treatment in T28 cells modulated their normal regulation of cell cycle progression. Nevertheless, no significant effects were observed for normal oral cells N28 (data not shown).

### The Effect of Taiwanin E on Cell Cycle-Related Proteins of Oral Cancer Cells

**Figure 3:**
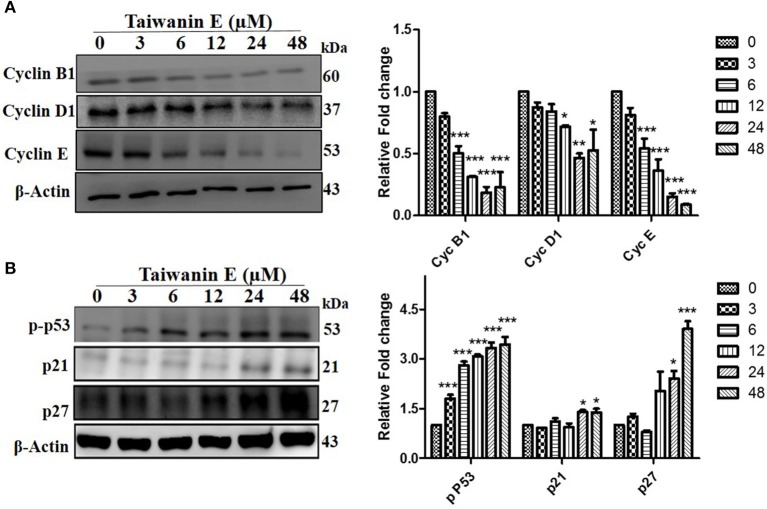
Effect of Taiwanin E on cell cycle-related proteins in oral cancer cells. T28 cells were treated with Taiwanin E (10 μM) for different time intervals and thereafter analyzed through Western blotting. Samples were assessed for the expression of Cyclin B1, Cyclin D1, and Cyclin E **(A)** and p53, p21, and p27 **(B)**, respectively. Graphs represent mean values of three independent experiment in T28 cells. *p < 0.05, **p < 0.01, and ***p < 0.001 represent significant differences compared with control.

We further investigated whether Taiwanin E affects cell cycle-related proteins in T28 oral cancer cells. To this end, cells were treated with Taiwanin E (10 μM) for different time periods from 0 to 48 h and thereafter analyzed through Western blotting. Interestingly, it was found that the cell cycle regulatory proteins, such as cyclin B1, cyclin D1, and cyclin E, were considerably decreased (Figure 3A), whereas p53, p21, and p27 protein levels were substantially increased following treatment with Taiwanin E (Figure 3B).

### The Effect of Taiwanin E on the Induction of Apoptosis in Oral Cancer Cells

**Figure 4:**
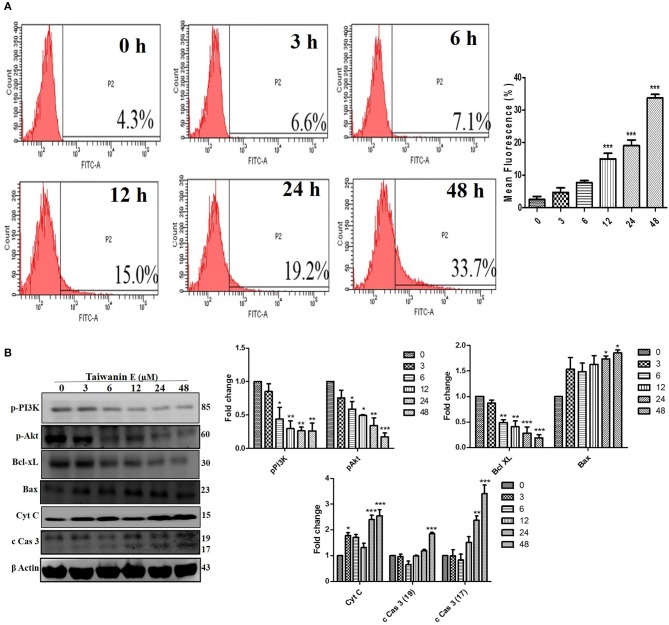
Effect of Taiwanin E on the induction of cellular apoptosis in oral cancer cells. T28 cells were treated with Taiwanin E (10 μM) for different time periods The samples were stained with a TUNEL kit and analyzed through flow cytometry **(A)**. T28 cells were treated with Taiwanin E (10 μM) for different time periods. Cell lysates were analyzed for the expressions of p-PI3K, p-Akt, Bcl-xL, Bax, Cyt C, and c Cas 3 through Western blotting **(B)**. Graphs represents the mean value of three separate experiment in the T28 cells. *p < 0.05, **p < 0.01, and ***p < 0.001 represent significant differences compared with control group.

We further investigated whether Taiwanin E could induce cellular apoptosis in T28 oral cancer cells. As evident through TUNEL assay, considerable apoptosis was observed following treatment with Taiwanin E (Figure 4A). To gain further insight into the effect of Taiwanin E on T28 oral cancer cells, we examined the protein levels of p-PI3K, p-Akt, Bcl-xL, Bax, cytochrome C, and cleaved caspase 3. Intriguingly, it was observed that T28 cells, when treated with Taiwanin E, showed a significant decrement in survival-related proteins, including p-PI3K, p-Akt, and anti-apoptotic protein Bcl-xL, in a time-dependent manner; contrastingly, the levels of Bax, Cyt C, and cleaved-caspase 3 were considerably decreased accordingly (Figure 4B).

### The Effect of Taiwanin E on the MAPK Signaling Cascade in Oral Cancer Cells

**Figure 5:**
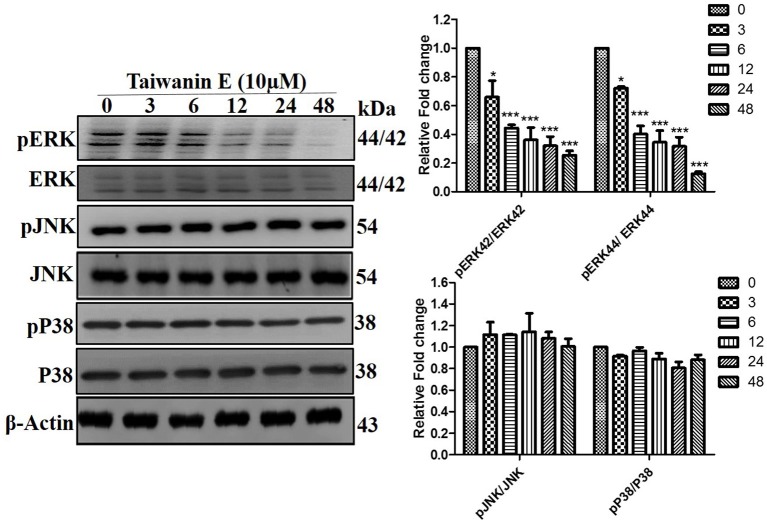
Taiwanin E modulate the ERK signaling cascade in oral cancer cells. T28 cells were treated with Taiwanin E (10 μM) for different time periods. Thereafter, cell lysates were analyzed for the expression of ERK, pERK, JNK, pJNK, p38, and p-p38 through Western blotting. Graphs represents mean values of three independent experiment in T28 cells. *p < 0.05 and ***p < 0.001 represent significant differences compared with control.

Further, the effect of Taiwanin E on the MAPK signaling cascade was investigated. As could be seen from the figure, following treatment with Taiwanin E, a considerable reduction in the p-ERK1/2 was observed; no significant effects, however, were observed for p-JNK and p-p38 proteins (Figure 5). Collectively, these results showed that Taiwanin E considerably regulated the MAPK signaling cascade.

### The Effects of Taiwanin E on ERK in Oral Cancer Cells

**Figure 6:**
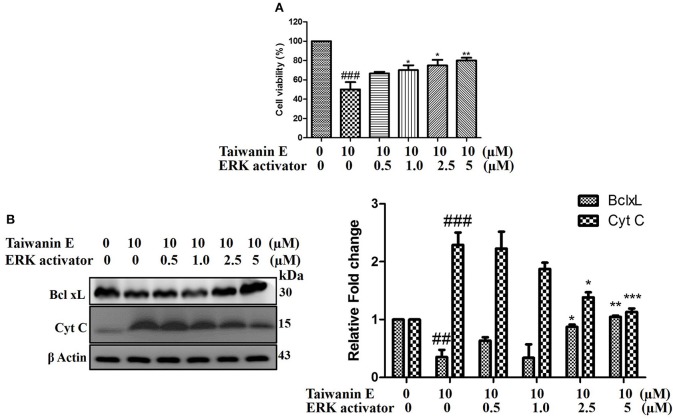
Effect of Taiwanin E on ERK activation in oral cancer cells. T28 cells were co-treated with ERK activator and Taiwanin E for 24 h. Thereafter, cell viability was analyzed through MTT assay **(A)**. T28 cells were co-treated with ERK activator and Taiwanin E for 24 h. Thereafter, cell lysates were analyzed through Western blotting for the expression of Bcl-xL and Cyt C proteins through Western blotting **(B)**. Graphs represent mean value of three separate experiment in T28 cells. ^*##*^*p* < 0.01 and _*###*_*p* < 0.001 represent significant differences compared with control group, whereas *p < 0.05, **p < 0.01, and ***p < 0.001 represent significant differences compared with the Taiwanin E-treated group.

To further investigate the underlying intricacies of modulation of ERK in the efficacy of Taiwanin E, the effect of the ERK activator on T28 cell proliferation was ascertained. Intriguingly, it was observed that ERK activator co-treatment with Taiwanin E in T28 cells considerably reversed the effect of Taiwanin E in a dose-dependent fashion (Figure 6A). In addition, we further examined whether the ERK activator co-treatment could interfere with the signal transduction pathway of cellular apoptosis. Interestingly, the results showed that co-treatment with the ERK activator considerably increased Bcl-xL protein levels and reduced Cyt C protein levels (Figure 6). These results highlighted the role of ERK in the efficacy of Taiwanin E against T28 oral cancer cells.

Collectively, it could be envisaged that Taiwanin E considerably modulated ERK, which eventually led to the inhibition of cancer cell survival, induced cell cycle arrest at G1, and apoptosis in T28 oral cancer cells. Collectively, the data convincingly demonstrated that Taiwanin E may potentially become an effective small molecular drug against oral cancer in the near future.

## Discussion

Various bioactive compounds (lignans, flavones, sesquiterpenoids, diterpenoids, cyclitols, steroids, etc.) derived from Taiwania has been shown to be effective against various debilitating diseases. Accumulating evidences have shown their efficacy against various forms of cancers, including A-549 lung carcinoma, MCF-7 breast adenocarcinoma, and HT-29 colon adenocarcinoma (22). In this research endeavor, we investigated the anti-cancer effect of Taiwanin E against arecoline and 4-nitroquinoline-1-oxide-induced OSCC and elucidated the underlying mechanism thereof. Basically, OSCC were treated with Taiwanin E and analyzed for their bioactivities against OSCC. Collectively, the data of the present study suggest that Taiwanin E significantly attenuated the cell viability of oral cancer cells (T28) in a dose- and time-dependent fashion. Moreover, it was observed that Taiwanin E induced G1 cell cycle arrest in T28 cells, as was evident through Flow cytometry studies, and further Western blot analysis suggested that Taiwanin E considerably downregulated cell cycle regulatory proteins, including Cyclin B1, Cyclin D1, and Cyclin E, and activated p53, p21, and p27 proteins, respectively. Furthermore, TUNEL staining showed that Taiwanin E induced considerable apoptosis in T28 cells, and, in addition, it was found that the cell survival proteins, such as p-PI3K and p-Akt and the anti-apoptotic protein Bcl-xL, were considerably reduced following treatment with Taiwanin E The pro-apoptotic proteins, such as Bax, Cyt C, and c Cas-3, were, however, considerably enhanced. Furthermore, in understanding the underlying intricacies mechanistically, it was found that Taiwanin E seemingly modulated ERK and thereby attenuated cell survival and induced cellular apoptosis in T28 oral cancer cells.

It is widely accepted that alteration in cell cycle progression causes severe cellular damage which eventually leads to apoptosis. It was found that Taiwanin E induces cell cycle arrest in OSCC in a dose and time dependent fashion. Analyzing the molecular intricacies underlying Taiwanin E-induced cell cycle arrest, it was observed that Taiwanin E treatment downregulated the expression of cell cycle regulatory proteins; in addition, Taiwanin E regulated the p53 tumor suppressor and increased the protein expression of p21 and p27. This is in concordance with the study by Shyur et al., wherein they demonstrated that Taiwanin A isolated from *Taiwania cryptomerioides* Hayata upregulated p53, phosphorylated p53, p21, and p27, and, contrastingly, downregulated the G(2)/M checkpoint mediators cyclins, leading to the induction of G(2)/M cell-cycle arrest in MCF-7 cells (13).

As a matter of fact, the apoptotic pathways are regulated through the orchestrated communication of diverse pro-apoptotic and anti-apoptotic signaling mediators. Essentially, the pro-apoptotic signaling mediators lead to the modulation of mitochondrial potential and subsequent release of mediators, which eventually triggers a cascade of events that lead to apoptosis. The data of the present study demonstrated that Taiwanin E treatment increased the amount of pro-apoptotic proteins. Bax released cytochrome C from the mitochondria, induced Caspase activation, and downregulated the survival protein p-PI3K, p-Akt, and anti-apoptotic Bcl-xL, which is certainly suggestive of their promising anti-cancer potential against OSCC.

MAPK/ERK signaling cascades are crucial pathways that plays an imperative role in the orchestration of normal cell proliferation, survival, and differentiation. It is widely accepted that aberrant regulation of MAPK has been involved in various human diseases, including cancer. Thus, the MAPK/ERK signaling network has been the focus of various research endeavors in order to ascertain novel target-based approaches for cancer therapeutics (28). Intriguingly, our study demonstrated that Taiwanin E modulated ERK signaling for therapeutic efficacy (28). This is in concordance with the reports, wherein authors have implicated the role of the ERK signaling mediator in the efficacy of the anti-cancer compounds. To this end, Wang et al. reported that Geraniin inhibited migration and invasion of human osteosarcoma cancer cells, seemingly through the regulation of the PI3K/Akt and ERK1/2 signaling pathways (29). Furthermore, Fong et al., while demonstrating the efficacy of BPIQ-induced cell migration and apoptosis in human non-small cell lung cancer (NSCLC) cells, highlighted that MAPK kinases, in particular ERK mediators, were responsible for their anti-cancer potential (30). In addition, Kuo et al. have explicitly demonstrated that the a related compound of Taiwanin E, i.e., a novel derivative of Taiwanin A, inhibited dual key proliferation signaling transduction pathways in a Triple-Negative Breast Cancer model (31).

Collectively, keeping in mind the fact that various phytochemicals, such as taxol (from *Taxus brevifolia*) and camptothecin derivatives (from *Camptotheca acuminate*), are widely explored for cancer treatment, and, as Taiwanin E displayed lower cytotoxicity against normal oral cells, it could be envisaged as a promising drug to treat oral cancer in the near future, thus warranting future investigation.

## Data Availability Statement

The datasets generated for this study are available on request to the corresponding author.

## Author Contributions

S-HW and C-YH contributed toward the conception and design of the study. S-HW, H-CW, and KB performed the experiments and data analysis. KB, C-JC, and C-YH contributed to the manuscript writing, editing, and proofreading. Y-HK, Y-PC, H-HH, D-TB, VV, Y-HC, and P-JL supported the experiment design, data analysis, and the manuscript writing and proofreading.

### Conflict of Interest

The authors declare that the research was conducted in the absence of any commercial or financial relationships that could be construed as a potential conflict of interest. The handling Editor declared a shared affiliation, though no other collaboration, with several of the authors C-YH and Y-HC.

## References

[1] Chrysant SG Chrysant GS. Herbs used for the treatment of hypertension and their mechanism of action. Curr Hypertension Rep. (2017) 19:77. 10.1007/s11906-017-0775-5 28921053

[2] Chiang JT Badrealam KF Shibu MA Cheng SF Shen CY Chang CF. Anti-apoptosis and anti-fibrosis effects of eriobotrya japonica in spontaneously hypertensive rat hearts. Int J Mol Sci. (2018) 19:E1638. 10.3390/ijms19061638 29857545 PMC6032044

[3] Hedigan K. Cancer: herbal medicine reduces chemotherapy toxicity. Nat Rev Drug Discov. (2010) 9:765. 10.1038/nrd3280 20885407

[4] Efferth T Li PC Konkimalla VS Kaina B. From traditional Chinese medicine to rational cancer therapy. Trends Mol Med. (2007) 13:353–61. 10.1016/j.molmed.2007.07.001 17644431

[5] Li-Weber M. Targeting apoptosis pathways in cancer by Chinese medicine. Cancer Lett. (2013) 332:304–12. 10.1016/j.canlet.2010.07.015 20685036

[6] Newman DJ Cragg GM. Natural products as sources of new drugs over the last 25 years. J Nat Prod. (2007) 70:461–77. 10.1021/np068054v 17309302

[7] Abdelfadil E Cheng YH Bau DT Ting WJ Chen LM Hsu HH. Thymoquinone induces apoptosis in oral cancer cells through p38beta inhibition. Am J Chinese Med. (2013) 41:683–96. 10.1142/S0192415X1350047X 23711149

[8] Chen MC Huang CY Hsu SL Lin E Ku CT Lin H. Retinoic acid induces apoptosis of prostate cancer DU145 cells through Cdk5 overactivation. Evid-Based Compl Altern Med. (2012) 2012:580736. 10.1155/2012/580736 23304206 PMC3532922

[9] Tsai KH Hsien HH Chen LM Ting WJ Yang YS Kuo CH. Rhubarb inhibits hepatocellular carcinoma cell metastasis via GSK-3-beta activation to enhance protein degradation and attenuate nuclear translocation of beta-catenin. Food Chem. (2013) 138:278–85. 10.1016/j.foodchem.2012.10.038 23265488

[10] Dung TD Chang HC Binh TV Lee MR Tsai CH Tsai FJ. Zanthoxylum avicennae extracts inhibit cell proliferation through protein phosphatase 2A activation in HA22T human hepatocellular carcinoma cells *in vitro* and *in vivo*. Int J Mol Med. (2012) 29:1045–52. 10.3892/ijmm.2012.938 22426520

[11] Dung TD Chang HC Chen CY Peng WH Tsai CH Tsai FJ. Zanthoxylum avicennae extracts induce cell apoptosis through protein phosphatase 2A activation in HA22T human hepatocellular carcinoma cells and block tumor growth in xenografted nude mice. Int J Mol Med. (2011) 28:927–36. 10.3892/ijmm.2012 21874223

[12] Ho PJ Chou CK Kuo YH Tu LC Yeh SF Taiwanin a induced cell cycle arrest and p53-dependent apoptosis in human hepatocellular carcinoma HepG2 *Cells Life Sci*. (2007) 80:493–503. 10.1016/j.lfs.2006.10.017 17182066

[13] Shyur LF Lee SH Chang ST Lo CP Kuo YH Wang SY. Taiwanin A inhibits MCF-7 cancer cell activity through induction of oxidative stress, upregulation of DNA damage checkpoint kinases, and activation of p53 and FasL/Fas signaling pathways. Phytomedicine. (2010) 18:16–24. 10.1016/j.phymed.2010.06.005 20637573

[14] Chang ST Cheng SS Wang SY. Antitermitic activity of essential oils and components from Taiwania (Taiwania cryptomerioides). J Chem Ecol. (2001) 27:717–24. 10.1023/a:1010397801826 11446295

[15] Chang ST Chen PF Wang SY Wu HH. Antimite activity of essential oils and their constituents from Taiwania cryptomerioides. J Med Entomol. (2001) 38:455–7. 10.1603/0022-2585-38.3.455 11372974

[16] Tsai CY Fang HY Shibu MA Lin YM Chou YC Chen YH. Taiwanin C elicits apoptosis in arecoline and 4-nitroquinoline-1-oxide-induced oral squamous cell carcinoma cells and hinders proliferation via epidermal growth factor receptor/PI3K suppression. Environ Toxicol. (2019) 34:760–67. 10.1002/tox.22742 30884126

[17] Hsieh CH Hsu HH Shibu MA Day CH Bau DT Ho CC. Down-regulation of beta-catenin and the associated migration ability by Taiwanin C in arecoline and 4-NQO-induced oral cancer cells via GSK-3beta activation. Mol Carcinogen. (2017) 56:1055–67. 10.1002/mc.22570 27648737

[18] Lin KH Shibu MA Kuo YH Chen YC Hsu HH Bau DT. Taiwanin C selectively inhibits arecoline and 4-NQO-induced oral cancer cell proliferation via ERK1/2 inactivation. Environ Toxicol. (2017) 32:62–9. 10.1002/tox.22212 26537528

[19] Murugan AK Munirajan AK Tsuchida N. Ras oncogenes in oral cancer: the past 20 years. Oral Oncol. (2012) 48:383–92. 10.1016/j.oraloncology.2011.12.006 22240207

[20] Silverman S Jr. Demographics and occurrence of oral and pharyngeal cancers. The outcomes, the trends, the challenge. J Am Dental Assoc. (2001) 132 (Suppl.):7s–11s. 10.14219/jada.archive.2001.0382 11803655

[21] Yu GP Mehta V Branovan D Huang Q Hashibe M Zhang ZF. Improved survival among patients with base of tongue and tonsil cancer in the United States. Cancer Causes Control. (2012) 23:153–64. 10.1007/s10552-011-9864-y 22101504

[22] Chang ST Wang DS Wu CL Shiah SG Kuo YH Chang CJ. Cytotoxicity of extractives from Taiwania cryptomerioides heartwood. Phytochemistry. (2000) 55:227–32. 10.1016/S0031-9422(00)00275-2 11142847

[23] Hsu HH Kuo WW Day CH Shibu MA Li SY Chang SH. Taiwanin E inhibits cell migration in human LoVo colon cancer cells by suppressing MMP-2/9 expression via p38 MAPK pathway. Environ Toxicol. (2017) 32:2021–31. 10.1002/tox.22379 27807932

[24] Chao CN Lai CH Badrealam KF Lo JF Shen CY Chen CH. CHIP attenuates lipopolysaccharide-induced cardiac hypertrophy and apoptosis by promoting NFATc3 proteasomal degradation. J Cell Physiol. (2019) 234:20128–138. 10.1002/jcp.28614 30980393

[25] Khan BF Hamidullah Dwivedi S Konwar R Zubair S Owais M. Potential of bacterial culture media in biofabrication of metal nanoparticles and the therapeutic potential of the as-synthesized nanoparticles in conjunction with artemisinin against MDA-MB-231 breast cancer cells. J Cell Physiol. (2018) 234:6951–64. 10.1002/jcp.27438 30443899

[26] Zhang X Bommareddy A Chen W Khalifa S Kaushik RS Fahmy H. Sarcophine-diol, a chemopreventive agent of skin cancer, inhibits cell growth and induces apoptosis through extrinsic pathway in human epidermoid carcinoma A431 Cells. Transl Oncol. (2009) 2:21–30. 10.1593/tlo.08190 19252748 PMC2647699

[27] He X Maimaiti M Jiao Y Meng X Li H. Sinomenine Induces G1-Phase Cell Cycle Arrest and apoptosis in malignant glioma cells via downregulation of sirtuin 1 and induction of p53 acetylation. Technol Cancer Res Treat. (2018) 17:1533034618770305. 10.1177/1533034618770305 29756546 PMC5952277

[28] Roberts PJ Der CJ. Targeting the Raf-MEK-ERK mitogen-activated protein kinase cascade for the treatment of cancer. Oncogene. (2007) 26:3291–310. 10.1038/sj.onc.1210422 17496923

[29] Wang Y Wan D Zhou R Zhong W Lu S Chai Y. Geraniin inhibits migration and invasion of human osteosarcoma cancer cells through regulation of PI3K/Akt and ERK1/2 signaling pathways. Anti-Cancer Drugs. (2017) 28:959–66. 10.1097/CAD.0000000000000535 28704237

[30] Fong Y Wu CY Chang KF Chen BH Chou WJ Tseng CH. Dual roles of extracellular signal-regulated kinase (ERK) in quinoline compound BPIQ-induced apoptosis and anti-migration of human non-small cell lung cancer cells. Cancer Cell Int. (2017) 17:37. 10.1186/s12935-017-0403-0 28286419 PMC5339964

[31] Kuo YH Chiang EI Chao CY Rodriguez RL Chou PY Tsai SY. Dual inhibition of key proliferation signaling pathways in triple-negative breast cancer cells by a novel derivative of Taiwanin A. Mol Cancer Therapeutics. (2017) 16:480–93. 10.1158/1535-7163.MCT-16-0011 27956520

